# The Impact of the Impella RP^®^ Device on a Failing Right Heart: A Modelling and Simulation Approach to Ascertain Its Potential

**DOI:** 10.3390/bioengineering13101105

**Published:** 2026-09-23

**Authors:** Beatrice De Lazzari, Angela Richter, Christoph Nix, Roberto Badagliacca, Annalisa Pitino, Mercedes Gori, Gianmarco Scoccia, Massimo Capoccia, Claudio De Lazzari

**Affiliations:** 1 Department of Movement, Human and Health Sciences, University of Rome “Foro Italico”, 00135 Rome, Italy; beatrice.delazzari@uniroma4.it; 2 Interuniversity Centre of Bioengineering of the Human Neuromusculoskeletal System, University of Rome “Foro Italico”, 00135 Rome, Italy; 3 J&J MedTech Heart Recovery, Neuenhofer Weg, 52074 Aachen, Germany; arichte2@its.jnj.com (A.R.); cnix1@its.jnj.com (C.N.); 4 Department of Clinical, Internal Anesthesiology and Cardiovascular Sciences, “Sapienza” University of Rome, 00185 Rome, Italy; roberto.badagliacca@uniroma1.it (R.B.); gianmarcoscoccia@gmail.com (G.S.); 5 National Research Council, Institute of Clinical Physiology (IFC-CNR), 00185 Rome, Italy; annalisa.pitino@cnr.it (A.P.); mercedes.gori@cnr.it (M.G.); 6 Major Arterial Centre, Glan Clwyd Hospital, Betsi Cadwaladr University Health Board, Rhyl LL18 5UJ, UK; capoccia@doctors.org.uk

**Keywords:** numerical model, right ventricular assist device, right ventricular dysfunction, haemodynamic parameters, external work, Impella RP^®^

## Abstract

Background: Indications for right ventricular assist device (RVAD) insertion include right heart failure after implantation of a left ventricular assist device or early graft failure following heart transplantation. This study aimed to investigate how the upstream and downstream circulatory network interacts with the Impella RP^®^ device. Methods: A numerical model of the Impella RP^®^ device was implemented within the CARDIOSIM^©^ software platform for this study. In the numerical configuration, the RVAD aspirated blood from either the right atrium (RA-PA connection) or the right ventricle (RV-PA connection) and delivered it to the pulmonary artery. Only RA-PA connection is the currently used setting for the Impella RP^®^ device in clinical practice. Based on right ventricular (RV) decompression and total flow, our study may help define the need for a direct RV-unloading Impella RP^®^. Results: The simulations showed that while activating the RVAD in RA-PA mode, regardless of its rotational speed, the mean pulmonary artery pressure (PAP) percentage change was higher than that in the unsupported condition when the mean systemic venous pressure (SVP) and the pulmonary artery wedge pressure (PAWP) were both set to 20 [mmHg]. When RV-PA connection was applied, a similar trend was observed, although the PAP percentage changes were approximately halved compared to those when the RA-PA connection was applied. Conclusions: The Impella RP^®^ device has the potential to become a valid option for RV support based on current experimental and simulation data. Although already in use, further evaluation in the clinical setting will likely confirm its potential and lead to a more routinely application for RV support.

## 1. Introduction

The prognostic role of the right ventricle (RV) should be considered in the context of left-sided heart failure [1]. Failure of systolic function adaptation leads to increased dimensions with a negative effect on diastolic ventricular interactions [2]. RV-PA coupling has significant reserve in the context of elevated RV afterload, although the level of uncoupling that leads to RV failure remains not completely defined [3]. Better understanding of the pathophysiology of RV failure may well help with its initial medical management and timing of mechanical circulatory support. Prolonged survival by effective medical treatment becomes the grounds for the development of right heart failure secondary to chronic left ventricular dysfunction. Patients remain compensated as long as the right ventricle is functional. The ability to track the RV based on better monitoring of afterload and functional reserve may help change the course of the disease before the RV reaches the threshold that may limit both medical and LVAD treatment [4]. Right ventricular failure remains quite a challenging condition to manage in view of its complex background and still incomplete understanding of its pathophysiology [5,6,7]. Indications for right ventricular assist device (RVAD) insertion include right heart failure after implantation of a left ventricular assist device (LVAD) or early graft failure following heart transplantation. Right ventricular pathophysiology in the setting of pulmonary hypertension [3] represents the prevalent pathological condition for RVAD insertion, whether to support a failing left ventricle or a primarily involved right ventricle [1]. The development of RV dysfunction in end-stage lung disease is related to acute or chronic RV afterload increase due to the underlying lung disease or hypercapnic/hypoxic respiratory failure. Initial management of acute RV dysfunction includes volume optimisation, afterload reduction (nitric oxide), inotropes to restore contractility (milrinone, dobutamine) and improvement of systemic perfusion (noradrenaline, adrenaline, vasopressin). The presence of pulmonary hypertension would require pulmonary vasodilators such as prostacycline, phosphodiesterase-5 inhibitors and endothelin receptor antagonists. Persistence of RV dysfunction leading to failure would be an indication for extracorporeal life support (ECLS). Although veno-venous extracorporeal membrane oxygenation (VV ECMO) can be considered an initial strategy in the presence of RV dysfunction, it does not provide RV support [8,9,10,11], requiring reconfiguration to veno-pulmonary ECMO (VP ECMO) [12] using the ProtekDuo (Pittsburgh, PA, USA), which is a single-site dual-lumen cannula for percutaneous insertion through the right internal jugular vein and placement of its tip in the main pulmonary artery [13,14]. The proximal fenestration of the cannula drains venous blood from the right atrium (RA) to the ECMO system, which is followed by re-infusion into the main pulmonary artery through the distal fenestrations. This configuration, namely dual-lumen (dl) V-P ECMO, bypasses the RV completely, leading to suitable and effective RV assistance [15]. The use of VP ECMO may reduce the development or progression of right ventricular dysfunction and lead to a potentially successful outcome [16,17,18,19].

Percutaneous and surgically implanted right ventricular assist devices (RVADs) have been investigated in different clinical settings [20]. We have previously focused our attention on the ProtekDuo [21], which involves a complete bypass of the right ventricle, leading to improved pulmonary flow, left atrial filling pressure and left ventricular preload with a reduced leftward shift in the interventricular septum observed in right ventricular failure [17,22].

Another option would be the use of the Impella 2.5 device, an axial flow pump that is retrogradely advanced through the aortic valve and works by aspirating blood from the left ventricle to forward it directly into the ascending aorta (LVAD). Although no longer available on the market, the device has been previously studied by our group [23,24].

The purpose of this study is to investigate the Impella RP^®^ device ’s potential for the support of a failing right ventricle and argue for its role in a clinical setting. In this study, we consider the following RV conditions:

- PH-adapted, describing successful right ventricular remodelling to handle increased pressure without failing, maintaining cardiac output and normal structure, usually with reversible changes;
- PH-maladapted, resulting in ventricular dilatation, dysfunction, and severe damage when the right ventricle cannot sustain the elevated pressure.

Only RA-PA connection is the currently used setting for the Impella RP^®^ device in a clinical context. Based on right ventricular (RV) decompression and total flow or TF (the sum of the pump flow and right ventricular output), the present work may reveal the need for the development of a direct RV-unloading Impella RP^®^ device.

## 2. Materials and Methods

The pressure–flow curves for the Impella RP^®^ Johnson & Johnson MedTech/Abiomed (Johnson, Irvine, CA, USA) right ventricular assist device (RVAD) plotted in Figure 1 are the starting point for the development of a numerical model of the right cardiovascular system and the Impella RP^®^ device pump to investigate their interactions and the performance of the device as an RVAD.

A new module incorporating a right circulatory network based on a time-varying elastance model was developed within the CARDIOSIM^©^ software (version 8.3.2) platform to replicate the behaviour of the right cardiovascular network when the Impella RP^®^ RVAD aspirates blood from the right atrium and delivers it to the pulmonary artery (RA-PA connection) or when the RVAD aspirates blood from the right ventricle and delivers it to the pulmonary artery (RV-PA connection). In this half-circuit configuration, the right atrium and ventricle are connected to the pulmonary circulation via an RLC network.

The potential of the Impella RP^®^ device to help patients with right ventricular failure could be assessed and defined according to this arrangement by controlling the systemic filling pressure (or systemic venous pressure) (SVP) and the pulmonary artery wedge pressure (PAWP) to appropriate levels.

The following equation for the instantaneous right ventricular pressure has been used to replicate the behaviour of the right ventricle:

(1) $$P_{rv}\left(t\right)=P_{rv,0}+e_{rv}\left(t\right)‧\left[V_{rv}^{*}\left(t\right)-V_{rv,0}\right]$$

where *P_rv_*(*t*) is the right ventricular instantaneous pressure, V*_rv_(*t*) is the instantaneous right ventricular free wall volume, *e_rv_*(*t*) is the right ventricular elastance, *V_rv_*_,0_ is the resting right ventricular volume and *P_rv_*_,0_ is the resting right ventricular pressure. The right ventricular elastance is described by the following:

(2) $$e_{rv}\left(t\right)=E_{rv,d}+\left(\frac{E_{rv,s}-E_{rv,d}}{2}\right)\cdot {\bar{e}}_{rv}(t)$$

where

(3) $${\bar{e}}_{rv}\left(t\right)=\left\{\begin{array}{c} 1-cos\left(\frac{t}{T_{T}}\cdot \pi \right)\;0\leq t\leq T_{T}\\ 1+cos\left(\frac{t-T_{T}}{T_{TE}-T_{T}}\cdot \pi \right)\;T_{T}<t\leq T_{TE}\\ 0\;T_{TE}<t\leq T \end{array}\right.$$

*T* is the duration of the ECG signal (Figure 2), *T_TE_* is the end of ventricular systole and *T_T_* is the *T*-wave peak time. *E_rv_*_,*d*_ and *E_rv_*_,*s*_ are the right diastolic and systolic ventricular elastance, respectively.

The following equation for the instantaneous right atrial pressure has been used to replicate the behaviour of the right atrium:

(4) $$P_{ra}\left(t\right)=P_{ra,0}+e_{ra}\left(t\right)‧\left[V_{ra}^{*}\left(t\right)-V_{ra,0}\right]$$

*P_ra_*(*t*) is the instantaneous right atrial pressure, [V*_ra_(*t*)] is the free wall volume, era is the right atrial elastance, *V_ra_*_,0_ is the resting right atrial volume and *P_ra_*_,0_ is the resting right atrial pressure.

The right atrial elastance is as follows:

(5) $$e_{ra}\left(t\right)=E_{ra,d}+\left(\frac{E_{ra,s}-E_{ra,d}}{2}\right)\cdot a_{ra}(t)$$

The activation function *a_ra_*(*t*) describing the contraction and the relaxation phases is as follows:

(6) $$a_{ra}\left(t\right)=\left\{\begin{array}{c} 0\;0\leq t\leq T_{PB}\\ 1-cos\left(\frac{t-T_{PB}}{T_{PE}-T_{PB}}\cdot 2\pi \right)\;T_{PB}<t\leq T_{PE}\\ 0\;T_{PE}<t\leq T \end{array}\right.$$

*E_ra_*_,*d*_ and *E_ra_*_,*s*_ are the right diastolic and systolic atrial elastance, respectively. According to the ECG, atrial systole is defined as the difference between the onset of atrial depolarization (*T_PB_*) and the end of atrial depolarization (*T_PE_*). The values of the parameter equations are listed in Appendix A.

Figure 3 shows the electrical analogue for the new right cardio-circulatory module within the CARDIOSIM^©^ platform. The picture reproduces the schematic representation of the Impella RP^®^ device used with RA-PA and RV-PA connection.

We used the following second-order polynomial to replicate the Impella RP^®^ device flow:

(7) $$Qpump=a\cdot \Delta P^{2}+b\cdot \Delta P+k$$

where Δ*P* is the pressure drop (Ventricular/Pump or Atrium/Pump) including all components from the inlet to outlet (so, any losses in the cannula are included), *Qpump* is the flow produced by Impella RP^®^ and finally, *a*, *b*, and *k* are reported in Table 1.

Figure 4, Figure 5 and Figure 6 show all the pressure–flow waveforms obtained using a quadratic equation (Equation (7)):

The configuration presented in Figure 3 allows changes in the mean systemic filling pressure (SVP), the pulmonary artery wedge pressure (PAWP) and, if necessary, the afterload in terms of pulmonary artery pressure (PAP). The afterload can be set to a specific value by activating an algorithm that maintains the mean PAP at the specified value by adjusting the pulmonary arterial resistance.

Setting the SVP and contractility with activation of the Impella RP^®^, it is possible to assess the effects on the right ventricular output flow rate, ventricular end systolic volume (ESV), end diastolic volume (EDV), total flow (TF) (the sum of the pump flow and right ventricular output) and PAP on the pulmonary arterial branch.

Setting the SVP and afterload and changing ventricular contractility allows the evaluation of the impact of changes in the rotational speed of the Impella RP^®^ device on the right ventricular output flow rate, ESV, EDV and TF. These simulations allow the assessment of the Impella RP^®^ device in different circulatory conditions either when the RVAD is applied with RV-PA or RA-PA connection. In addition, the PAWP can be set to a constant value.

We took the following steps to investigate the effects of the Impella RP^®^ device on several haemodynamic and energy parameters under different cardiovascular conditions:

- To reproduce a PH-adapted condition, the afterload (PAP) and the mean systemic venous pressure (SVP) was set at 60 and 20 [mmHg], respectively; the PAWP was set to 20 [mmHg]. An algorithm that could automatically change Rap and Cap was implemented in CARDIOSIM^©^ to maintain a constant PAP at 60 [mmHg]. In this first step, only Rap’s automated version had been switched on.
- Starting from the PH-adapted condition, the RVAD was applied with both RV-PA and RA-PA connection modes at different rotational speeds to evaluate the effects induced on right ventricular end diastolic/systolic volume (EDV/ESV), TF, right ventricular external work (EW), right ventricular pressure–volume area (PVA), and Ees/Ea ratio (right ventricular elastance/pulmonary arterial elastance).
- In the subsequent steps, the systemic venous pressure (SVP) was set to 10 and 15 [mmHg]; the PAWP was set to 10 and 15 [mmHg]. The algorithm maintained the PAP fixed at 60 [mmHg]. Systemic venous resistance was set at 30 [mmHg·cm^−3^·s]. Under these conditions, the Impella RP^®^ device was activated to aspirate blood at different rotational speeds from the right ventricle and atria, respectively.

Right ventricular remodelling in pulmonary hypertension consists of adaptation followed by exhaustion. In its early adaptive phase, the right ventricle responds appropriately in terms of hypertrophy and enhanced contractility to maintain forward flow and protect the left ventricle. Subsequently, persistent pressure overload overcomes the compensatory response, leading to maladaptive changes in terms of right ventricular dilatation, reduced systolic function and increased right-sided pressures. Therefore, we have considered PH-adapted and PH maladapted conditions during our simulations to take into account the whole spectrum of right ventricular remodelling. The acronym PH stands for pulmonary hypertension.

## 3. Results

To simulate a PH-adapted scenario, PAP and SVP were set to 60 and 20 [mmHg], respectively, with PAWP set to 20 [mmHg]. An algorithm that automatically changed the Rap value was activated to keep a constant PAP at 60 [mmHg]. The pulmonary arterial compliance (Cap) remained at 4.8 [cm^3^·mmHg^−1^]. The right ventricular elastance also remained at 0.5 [cm^−3^·mmHg]. The heart rate (HR) remained constant at 80 bpm. Figure 7 displays a CARDIOSIM^©^ screenshot for the PH-adapted conditions in which the right ventricular output flow is 3.65 [L/min] and the Ees/Ea is 1.32.

Figure 8 shows a PH-maladapted condition obtained from setting the right ventricular elastance to 0.5 [mmHg·cm^−3^], the PAWP and SVP both to 20 mmHg, the constant PAP at 60 [mmHg] and finally the Impella RP^®^ device applied both in RV-PA and RA-PA mode at 22,000 rpm (Figure 1).

The RVAD reduced the right ventricular end systolic volume (ESV) from 137.21 (Figure 7) to 130.98 [mL] when it was applied in RV-PA mode. If the pump was connected in RA-PA mode, the ESV would be 136.65 [mL] (Figure 8). In comparison to the unsupported condition, the EW (PVA) decreased by 10.57% (4.29%) when the Impella RP^®^ device was activated in RA-PA mode and by 17.22% (10.26%) when the pump was used in RV-PA mode. The TF increased by 8.22% when the assistance was activated in RV-PA mode and by 21.64% when it was applied in RA-PA mode. When the RVAD was activated in RA-PA (RV-PA) mode, the Ees/Ea ratio decreased by 9.85% (5.30%) compared to unsupported settings.

The right ventricular end diastolic volume (EDV) decreased from 182.83 (Figure 7) to 160.9 [mL] when the RVAD was applied in RV-PA mode at 28,000 rpm (Figure 9). Alternatively, the EDV was 171.36 [mL] when the pump was attached in RA-PA mode. Impella RP^®^ reduced the right ventricular end systolic volume (ESV) from 137.21 (Figure 7) to 126.38 [mL] when it was applied in RV-PA mode; the RA-PA connection would give an ESV of 135.68 [mL] (Figure 9).

The Impella RP^®^ device flow rate was 2.54 (2.89) [L/min] when the RA-PA (RV-PA) connection was selected, whereas the TF was 5.4 (Figure 9) and 4.28 [L/min], respectively (Figure 9). When the RVAD was activated in RA-PA (RV-PA) mode, the Ees/Ea ratio was 20.4% (18.9%) compared to unsupported settings.

In comparison to the unassisted condition, the EW (PVA) decreased by 22.36% (9.63%) when RVAD was activated in RA-PA mode and by 38.37% (21.26%) when the pump was used with RV-PA connection. When Impella RP^®^ was applied in RV-PA mode, the overall blood flow increased by 17.26%, and when it was activated in RA-PA mode, it increased by 47.95%. The right ventricular pressure–volume loop can be seen in panel [A] (Figure 9) for both unassisted (black line) and assisted mode with the RVAD applied in RV-PA (red line) and in RA-PA (blue line) connection. Similarly, the Impella RP^®^ device applied in RV-PA (red line) and in RA-PA (blue line) mode can be seen in both unassisted (black line) and assisted mode in the right atrial pressure–volume plane (loops in panel B). Impella RP^®^ activation reduces the internal area of the atrial loop, particularly in the right atrium–pulmonary artery connection (blue line).

When the RVAD was used in RV-PA connection and activated at a 33,000 rpm speed, the right EDV (ESV) decreased from 182.83 (137.2) to 150.8 (122.0) [mL] (Figure 10).

The right EDV (ESV) decreased from 182.83 (137.2) to 150.82 (122.0) [mL] when the Impella RP^®^ device was applied in RV-PA mode (Figure 10). Alternatively, the EDV (ESV) decreased by 166.38 (134.82) [mL] when the RVAD was used in RA-PA connection. When the RA-PA (RV-PA) assistance was activated, the Impella RP^®^ device flow rate was 3.67 (3.95) [L/min], while the TF was 6.2 (Figure 10) and 4.57 [L/min], respectively.

In the following simulations, SVP and PAWP were set both to 20 [mmHg]; PAP was not constant; systemic venous resistance was set to 60 [mmHg·cm^−3^·s]; the right ventricular elastance was set to 0.5 [mmHg·cm^−3^] and the pulmonary arterial compliance (Cap) was set to 4.8 [cm^3^·mmHg^−1^].

Figure 11 shows that the percentage change in mean pulmonary artery pressure (PAP) from the baseline is more pronounced when the Impella RP^®^ device aspirates blood from the atrium compared to when it aspirates from the ventricle. Additionally, both right ventricular assist device (RVAD) configurations result in a decrease in the ratio Eas/Ea when the mean PAP is not maintained at a constant level.

Under the same conditions (Figure 11), a reduction in EDV and ESV can be observed when Impella RP^®^ draws blood from the right ventricle (top left right panel respectively), while an increase in ESV is observed when the assist draws blood from the right atrium (top right panel), because the PAP increases and the native ventricle is not able to eject.

A new set of simulations was conducted with the SVP and PAWP set to 15 [mmHg], the systemic venous resistance set to 30 [mmHg cm^−3^·s], and the mean PAP maintained at a constant value of 60 [mmHg]. In this case, the Impella RP^®^ device was used at different rotational speeds in both RV-PA and RA-PA configurations. Figure 12 shows the percentage variations in right ventricular end diastolic volume (left panel) and right end systolic volume (right panel) obtained when the Impella RP^®^ device was activated in RV-PA and in RA-PA mode with 22,000, 28,000 and 33,000 rpm.

The Impella RP^®^ device working in both settings produces a percentage reduction in both ESV and EDV. When RVAD is connected in RV-PA mode, the percentage reduction is more evident; in this scenario, EDV reduces from approximately 6% (22,000 rpm) to approximately 18% (33,000 rpm), and ESV decreases from about 5% (22,000 rpm) to approximately 14% (33,000 rpm). There is little impact on ESV when Impella RP^®^ is turned on in RA-PA mode at different rotational speeds (Figure 12).

Figure 13 presents the percentage variations in TF and Ees/Ea (left panel) as well as EW and PVA (right panel) related to the starting conditions, for various activations of the Impella RP^®^ device configured in RV-PA and in RA-PA modes at different rotational speeds. Higher reduction in EW and PVA is observed when the RVAD aspirates blood from the right ventricle. Under these cardiovascular settings, the ratio Ees/Ea decreases by around 29% when the RVAD (33,000 rpm) aspirates blood from the atria and to approximately 30% when it drains blood from the ventricle.

In the following simulations, SVP and PAWP were both set to 10 mmHg; PAP was kept constant at 60 [mmHg]; systemic venous resistance was set to 30 [mmHg·cm^−3^·s] (Figure 14). In this case, the Impella RP^®^ device was used at different rotational speeds in both RV-PA and RA-PA connections.

Figure 14 shows the percentage variations in right ventricular end diastolic volume (left upper panel) and right end systolic volume (right upper panel) observed when the Impella RP^®^ device was activated in RV-PA and in RA-PA mode at 22,000, 28,000 and 33,000 rpm. EDV (ESV) was reduced by approximately (more than) 50% when RVAD aspirated blood from the right ventricle at a 33,000 rpm speed. The lower-left panel shows the percentage changes in right ventricular external work and pressure volume area (EW and PVA). Both EW and PVA decrease more when the RVAD aspirates blood from the right ventricle (left lower panel). The ratio Ees/Ea decreases by about 48% when the RVAD aspirates blood from the atria, but it increases by about 90% when it drains blood from the ventricle (right lower panel).

## 4. Discussion

The relationship between structure and function remains an essential element in clinical decision-making, which has to take into account how to address the disease and restore normality to a certain degree. The onset of right ventricular dysfunction should be considered in the context of pressure and volume overload or the primary myocardial disease [25,26]. The management of right heart failure is still difficult because of the complex geometry of the right heart and a lack of specific treatments aimed at recovery of right ventricular function, quite often leading to poor clinical outcome. The use of VV ECMO may have an initial role to play in the treatment of right heart failure, although its progression would require a more aggressive treatment, such as conversion to VP ECMO using the ProtekDuo, which is a single-site dual-lumen cannula inserted through the right internal jugular vein leading to complete bypass of the right ventricle [15,16,17,18,19] due to RA-PA configuration. Our previous simulation work has confirmed the effective haemodynamic assistance provided by ProtekDuo, as observed in the acute clinical setting [21]. We have also addressed the role of the Impella 2.5 device for left ventricular support, either alone or combined with the intra-aortic balloon pump [23,24]. This has given us the motivation to further investigate the haemodynamic effects of the Impella RP^®^ device for right ventricular support. The key element of this work is the significantly high correlation between the experimental and simulated pressure–flow curves, as shown in Figure 4, Figure 5 and Figure 6. This has given us the opportunity to develop realistic simulations for the evaluation of the effects of the Impella RP^®^ device on a failing right heart. The numerical model implemented within the CARDIOSIM^©^ software platform enabled us to study the implications of a possible evolution of the Impella RP^®^ device with a view to drain blood from the right ventricle and eject it into the pulmonary artery (RV-PA configuration). The simulation outcome shows that the Impella RP^®^ device seems to perform better when connected in RV-PA mode, with particular reference to the unloading of the right ventricle, EW, PVA and Ees/Ea leading to a more adequate ventriculo-arterial coupling. In fact, the RV-PA connection would decrease EW, PVA and Ees/Ea by 38.37%, 21.26% and 18.94% whilst the RA-PA connection would give 22.36%, 9.63% and 20.45% reduction. When both configurations are considered at different rotational speeds, RV-PA connection gives higher right ventricular unloading. More specifically, the RV-PA connection would reduce EDV and ESV by 18% and 14% whilst the RA-PA connection would give only 9% and 2% reduction. If we consider the right ventricular P-V loop, then a higher leftward shift is observed when the device is connected in RV-PA mode (Figure 9). The RV-PA connection would also have a lower effect on the mean pulmonary artery compared to the RA-PA connection (Figure 11). Nevertheless, the total flow is higher when support is delivered with RA-PA connection (Figure 13 and Figure 14). Percutaneous insertion through the right internal jugular vein would make its use even more appealing given the potential for patient’s mobilisation as observed with the ProtekDuo device.

The results for RV-PA can be provided as a sensitivity analysis for the fixed-wedge settings, which highlights a limitation of the network architecture selected for this work. Because of this limitation, the outcomes might lead to an overstatement of the true benefits of direct RV drainage.

Based on our findings, there are grounds for the development of an Impella RP^®^ device with the option of RA-PA and RV-PA connection, which would make it the device of choice for right ventricular support. Suitability for each connection would have to be considered according to the needs of each group of patients. Early intervention remains the best option for treating right heart failure and avoiding permanent damage.

## 5. Conclusions

Despite the limitations of the current simulation settings in terms of parameter overestimation, the correlation between experimental and simulation data shows that the Impella RP^®^ device has the potential to become a valid option for right ventricular support based on current experimental and simulation data. Although already in use, further evaluation in the clinical setting may confirm its potential and lead to its more routine application for right ventricular support. The potential evolution of the Impella RP^®^ device or a similar RVAD, with the option of both RA-PA and RV-PA connection, may lead to the development of a more adaptable device for the assistance of a failing right ventricle.

## Figures and Tables

**Figure 1:**
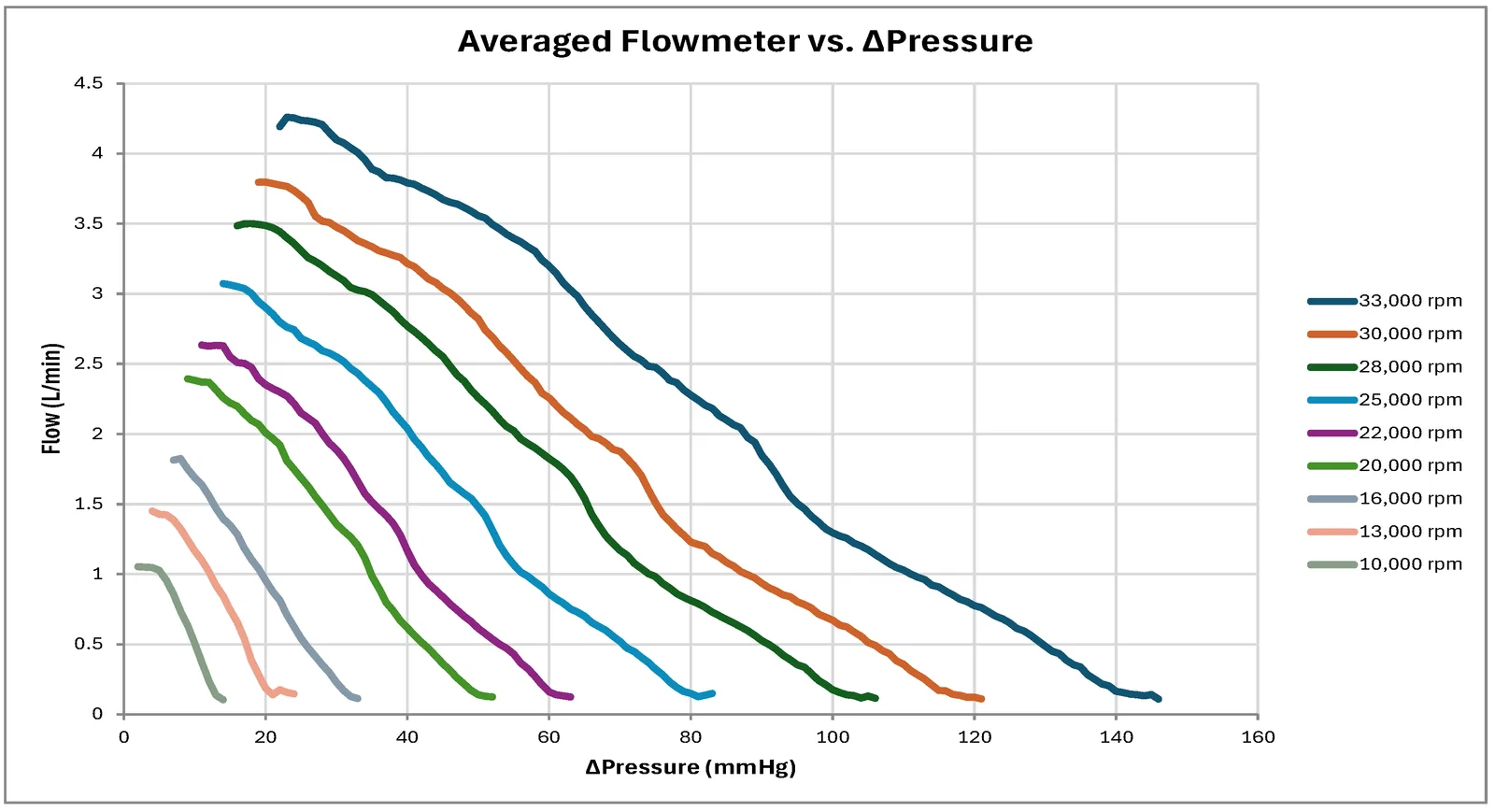
Pressure–flow curve for the Impella RP^®^ right ventricular assist device (RVAD) according to experimental data. *Δ*P is the pressure drop including all components from in-to outlet. Therefore, any losses in the cannula are included.

**Figure 2:**
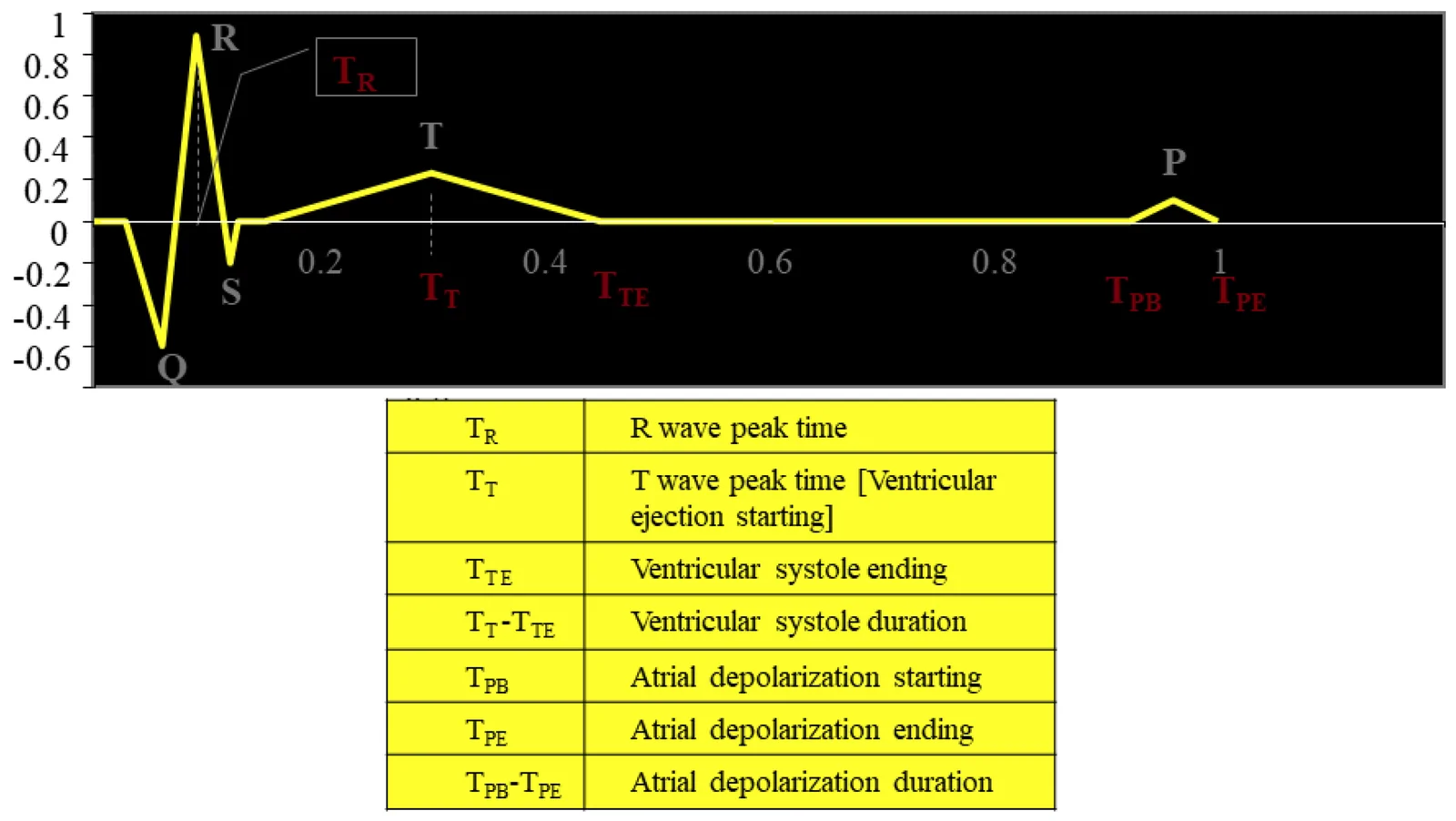
Schematic representation of the ECG signal.

**Figure 3:**
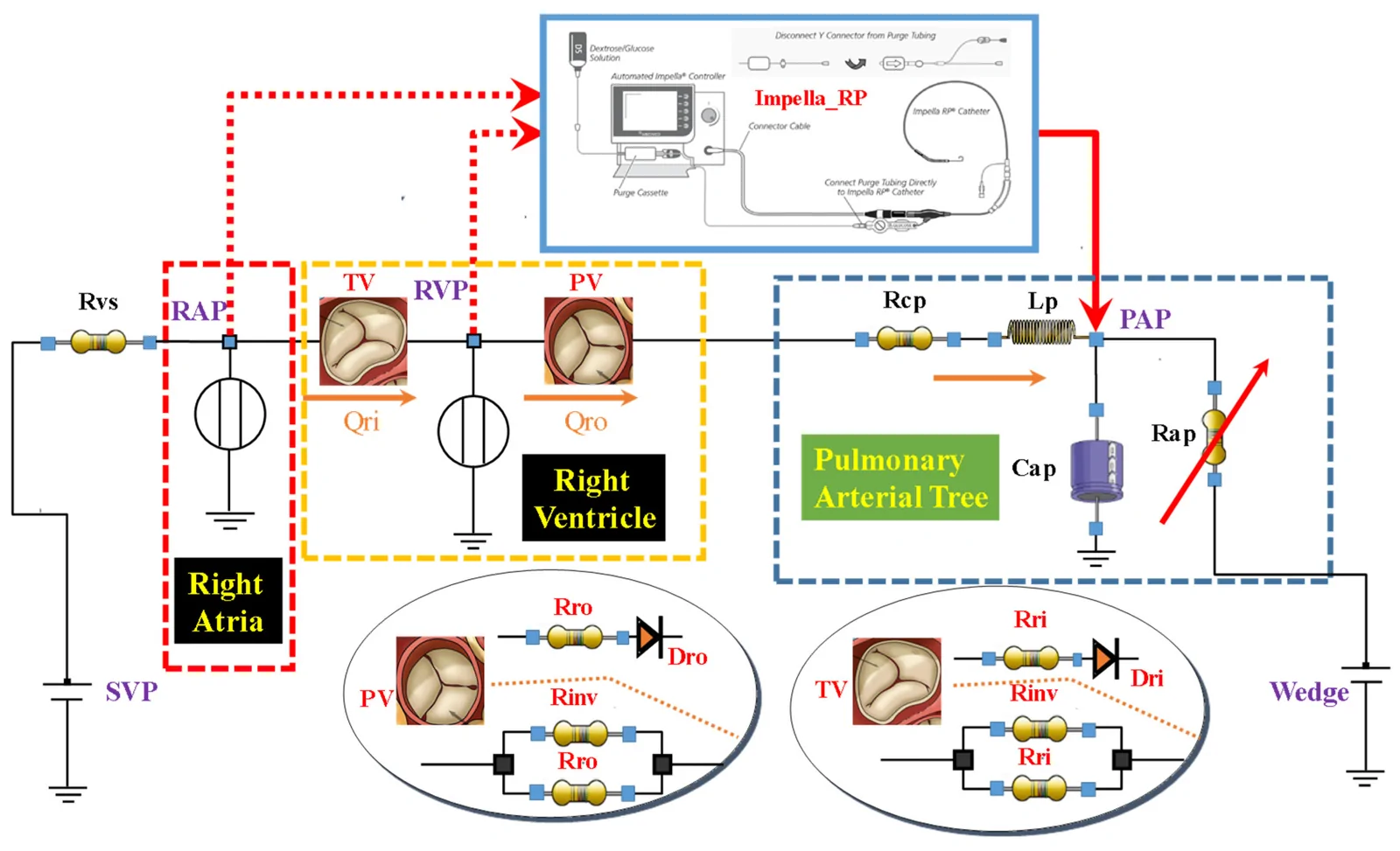
Open network that mimics the right cardiovascular system. The whole pulmonary circulatory system can be simulated using pulmonary characteristic resistance (Rcp), pulmonary arterial inductance (Lp), pulmonary arterial compliance (Cap), and varying pulmonary arterial resistance (Rap). Mean pulmonary arterial pressure (PAP), mean systemic venous pressure (SVP) and pulmonary artery wedge pressure (PAWP) can be set to constant settings.

**Figure 4:**
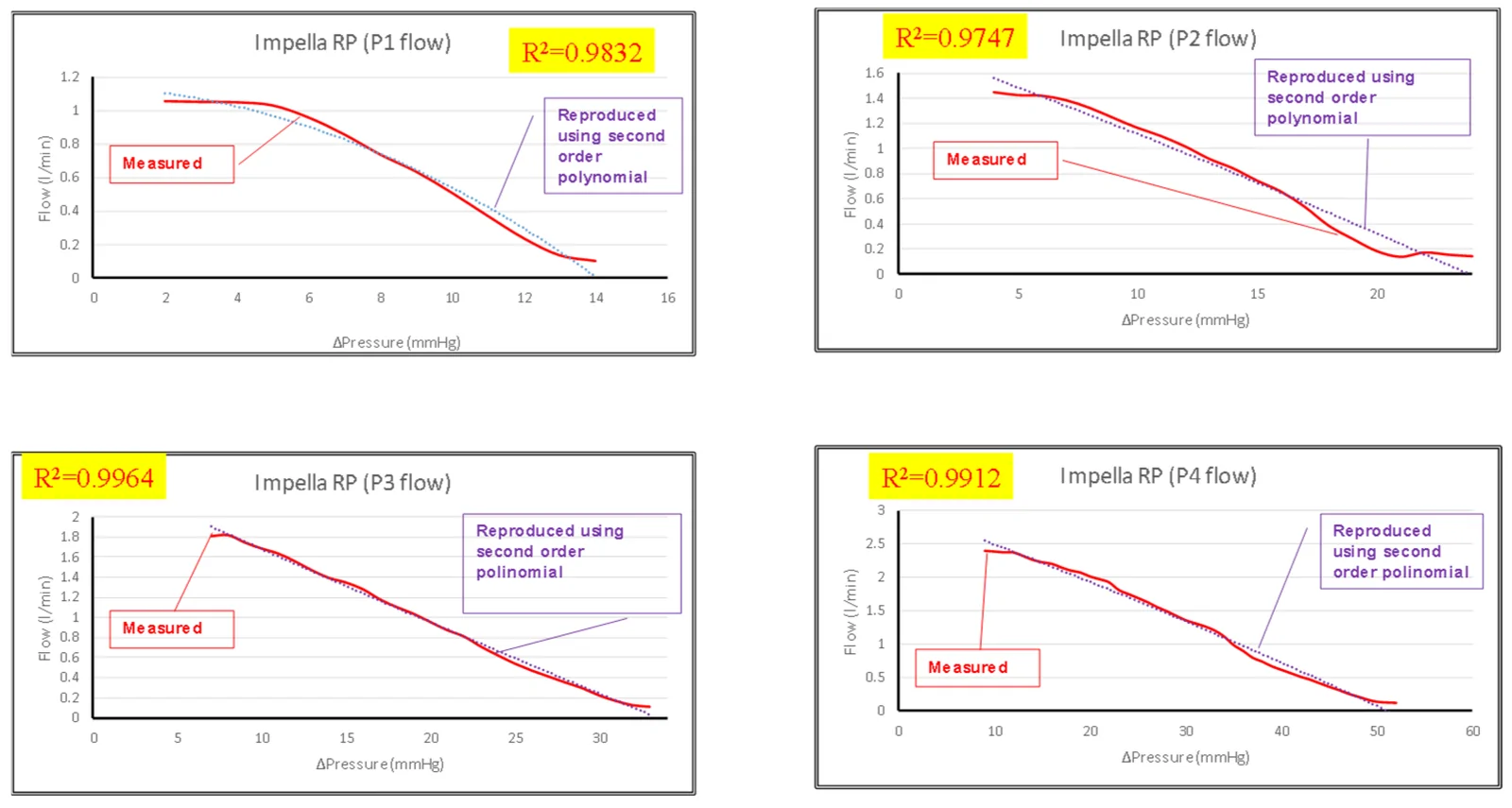
Comparison of values obtained mathematically using a second-order polynomial with measured values.

**Figure 5:**
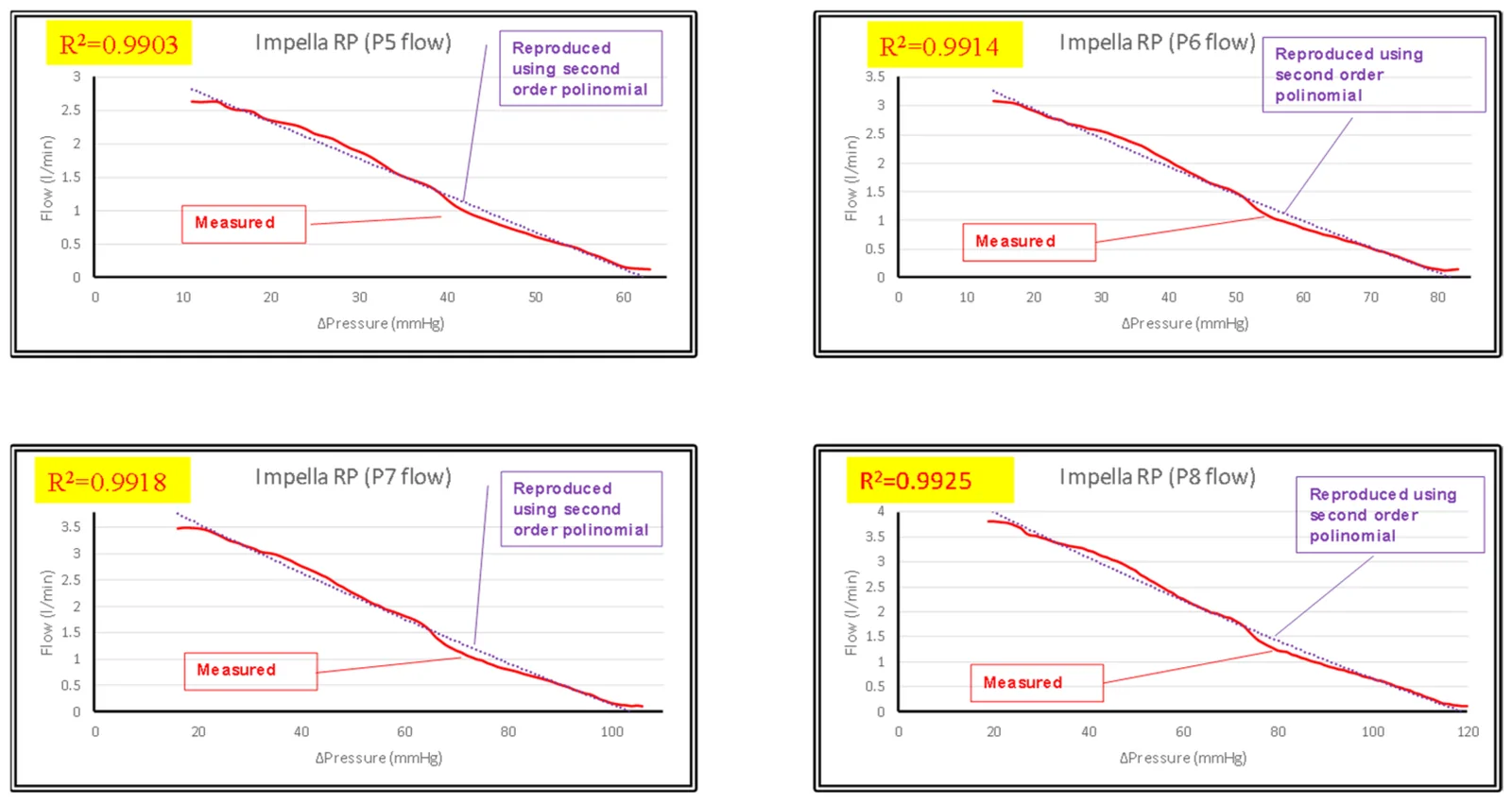
Comparison of values obtained mathematically using a second-order polynomial with measured values.

**Figure 6:**
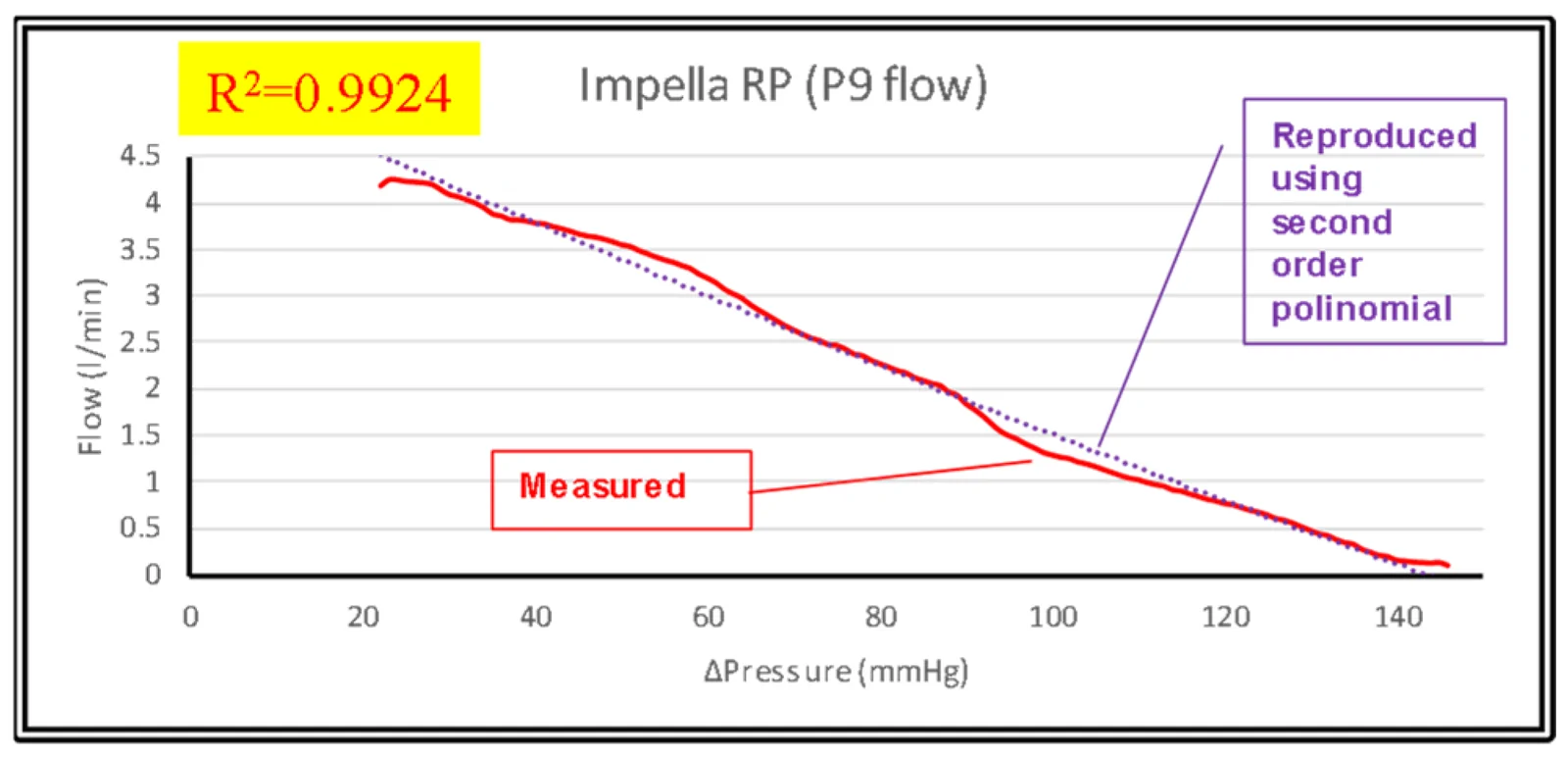
Comparison of values obtained mathematically using a second-order polynomial with measured values.

**Figure 7:**
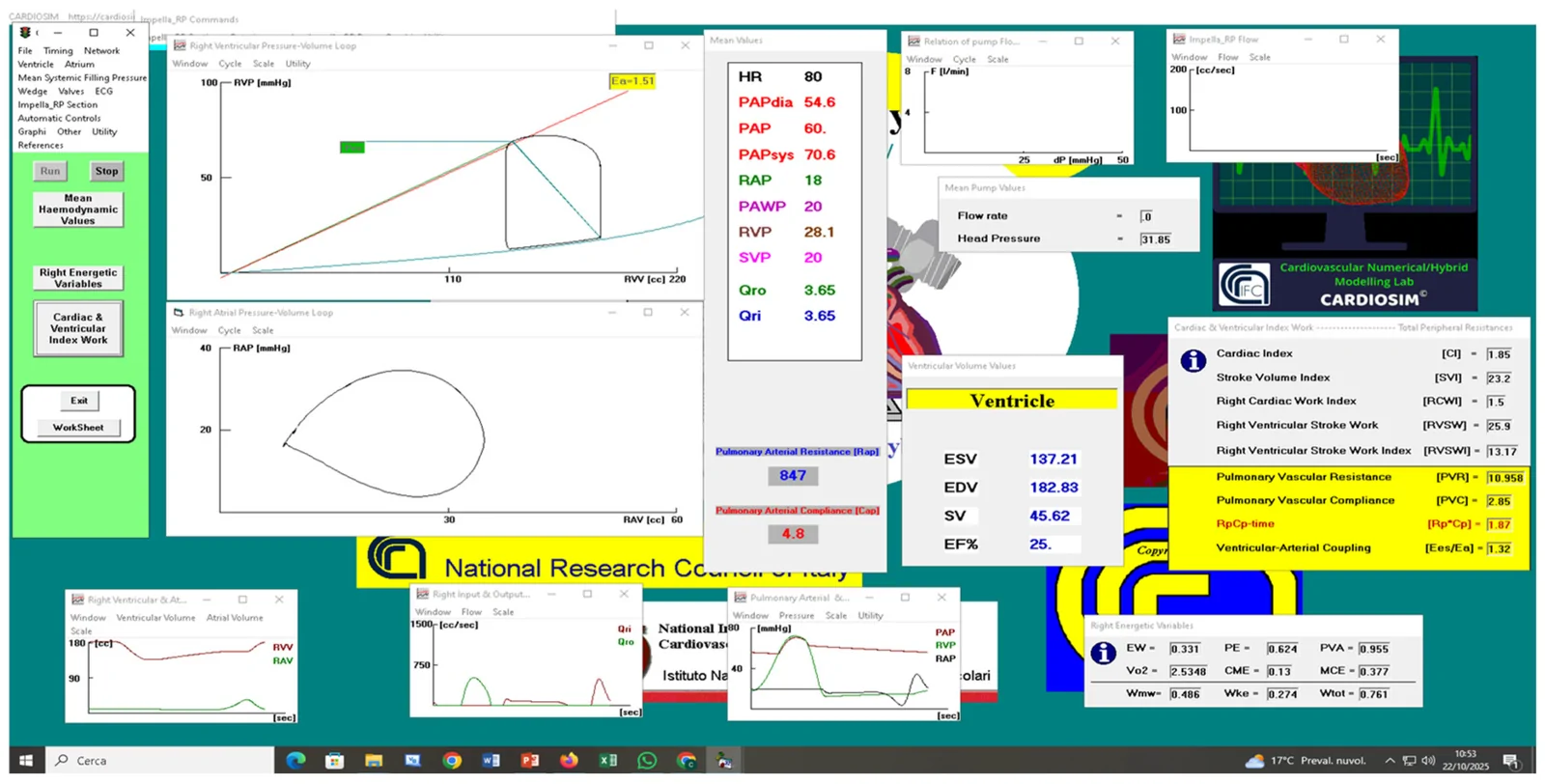
Screenshot of PH-adapted condition, setting the wedge to 20 [mmHg]. PAP was kept constant at 60 [mmHg] by automatically varying the Rap. Systemic venous pressure (SVP) was set to 20 [mmHg]. RVP is the right ventricular pressure. Qri and Qro are the input and output flow rates, respectively. The pulmonary arterial compliance (Cap) was set to 4.8 [cm^3^·mmHg^−1^]. The right ventricular elastance was set to 0.5 [cm^−3^·mmHg]. HR was set to 80 bpm.

**Figure 8:**
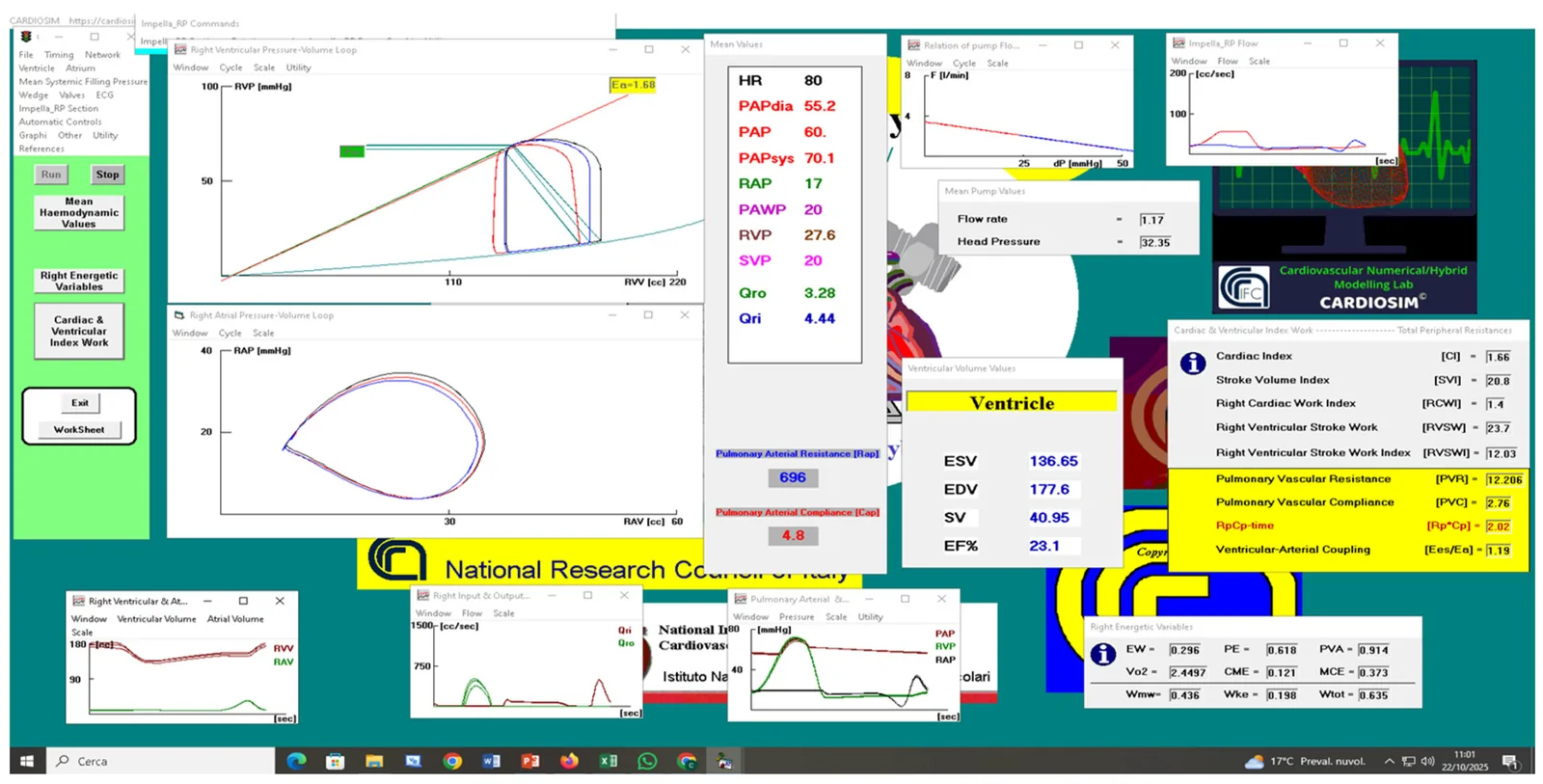
Screenshot of PH-maladapted condition, setting the wedge and SVP both to 20 [mmHg]. PAP was kept constant at 60 [mmHg] by automatically varying only the Rap. The right ventricular elastance was set to 0.5 [mmHg·cm^−3^]. The pulmonary arterial compliance (Cap) was set to 4.8 [cm^3^·mmHg^−1^]. Impella RP^®^ at 22,000 rpm was connected in RV-PA (red loop) and RA-PA mode (blue loop). Reported mean values show the effects induced by the RVAD connected in RA-PA mode. RVP (RAP) is the right ventricular (atrial) pressure and EW (PVA) is the right ventricular external work (pressure–volume area). The RVAD flow rate was 1.59 [L/min] when Impella RP^®^ was applied in RV-PA mode.

**Figure 9:**
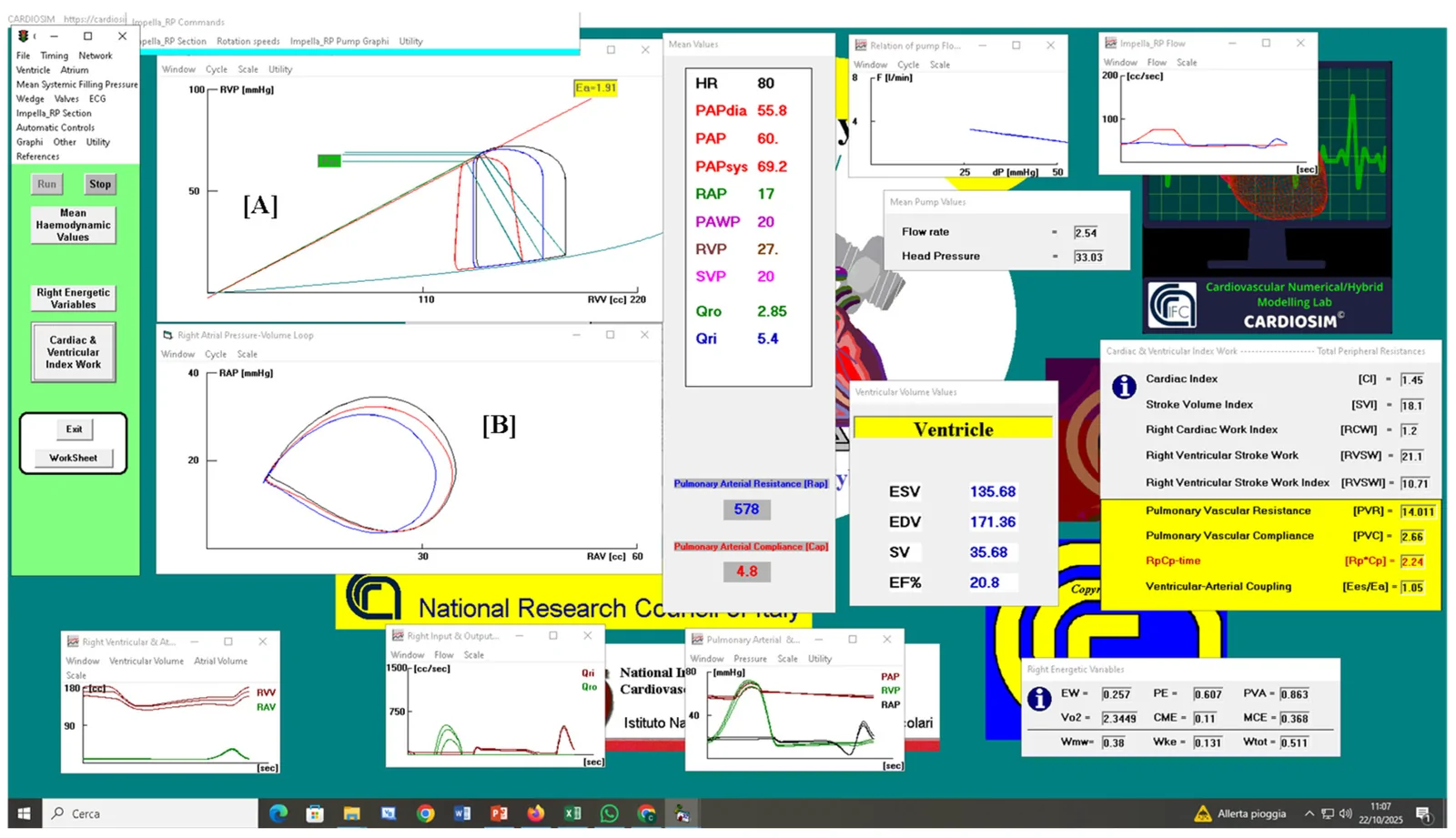
Screenshot of PH-maladapted condition setting PAWP and SVP both to 20 [mmHg]. PAP was kept constant at 60 [mmHg] by automatically varying only the Rap. The right ventricular elastance was set to 0.5 [mmHg·cm^−3^]. The pulmonary arterial compliance (Cap) was set to 4.8 [cm^3^·mmHg^−1^]. Impella RP^®^ with 28,000 rpm speed was connected in RV-PA (red loop) and RA-PA mode (blue loop). Reported mean values show the effects induced by the RVAD connected in RA-PA mode. The RVAD flow rate was 2.89 [L/min] when Impella RP^®^ was applied in RV-PA mode.

**Figure 10:**
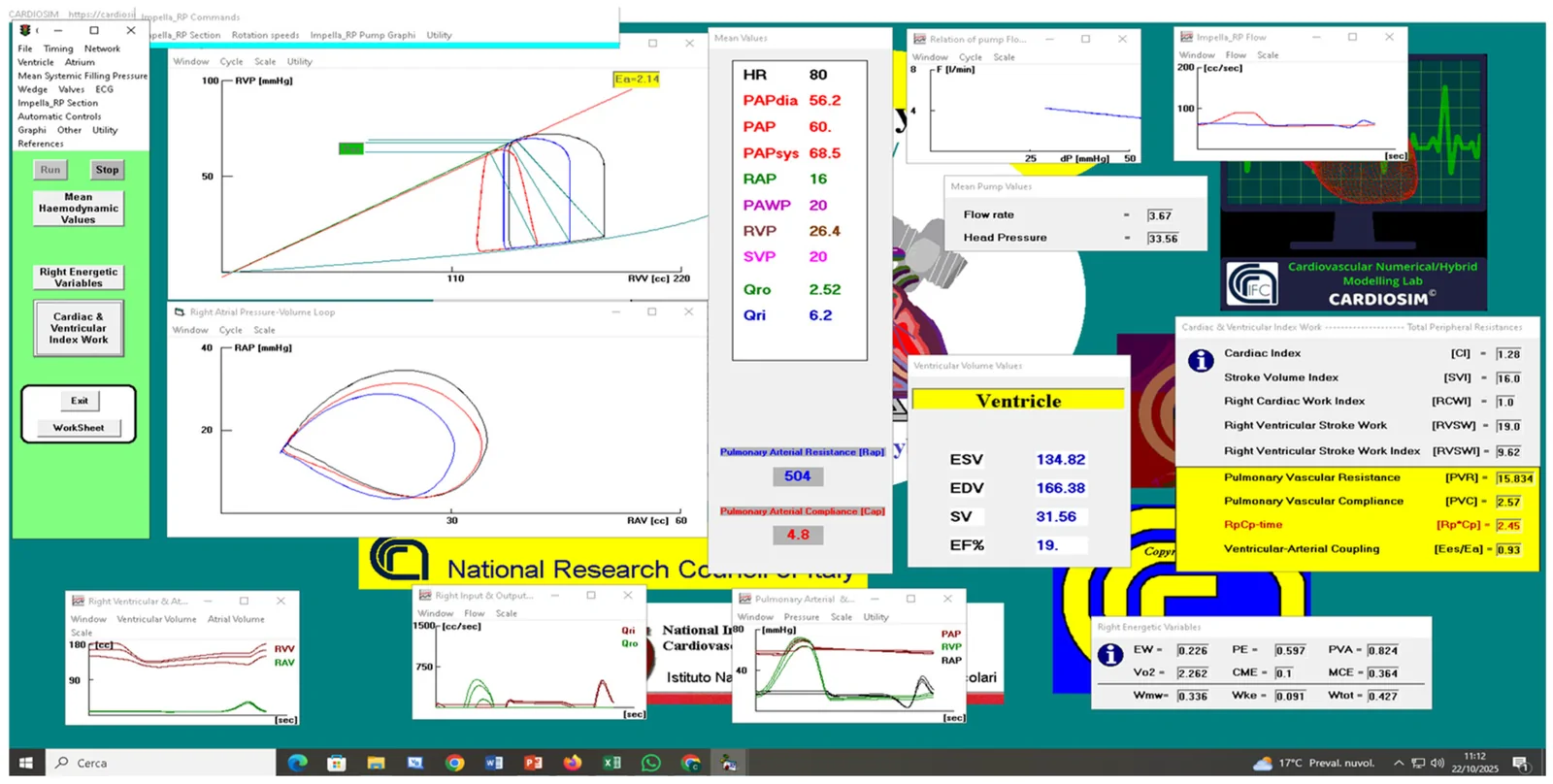
Screenshot of a PH-maladapted condition setting with the Impella RP^®^ device at a 33,000 rpm speed. The RVAD blood flow rate was 3.95 [L/min] when applied in RV-PA mode. RV-PA (red loop) and RA-PA connection (blue loop).

**Figure 11:**
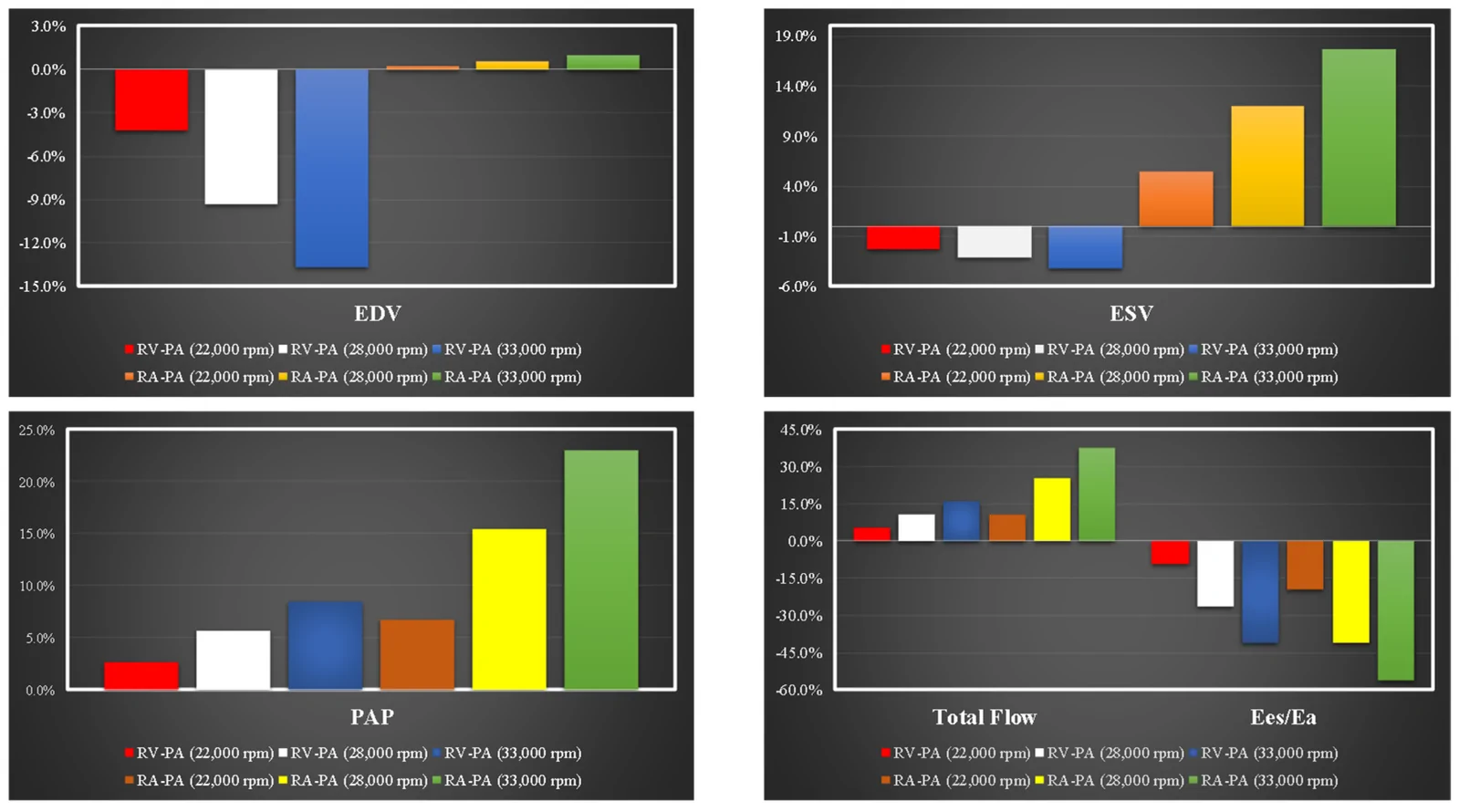
Percentage variations in right ventricular end diastolic volume (**top left** panel) and right ventricular end systolic volume (**top right** panel) in relation to the following starting conditions: both systemic venous pressure (SVP) and PAWP were kept constant at 20 [mmHg]. Systemic venous resistance is fixed at 60 [mmHg·cm^−3^·s]. Bottom left (**right**) panel shows percentage variations in mean PAP (Total Flow and Ees/Ea).

**Figure 12:**
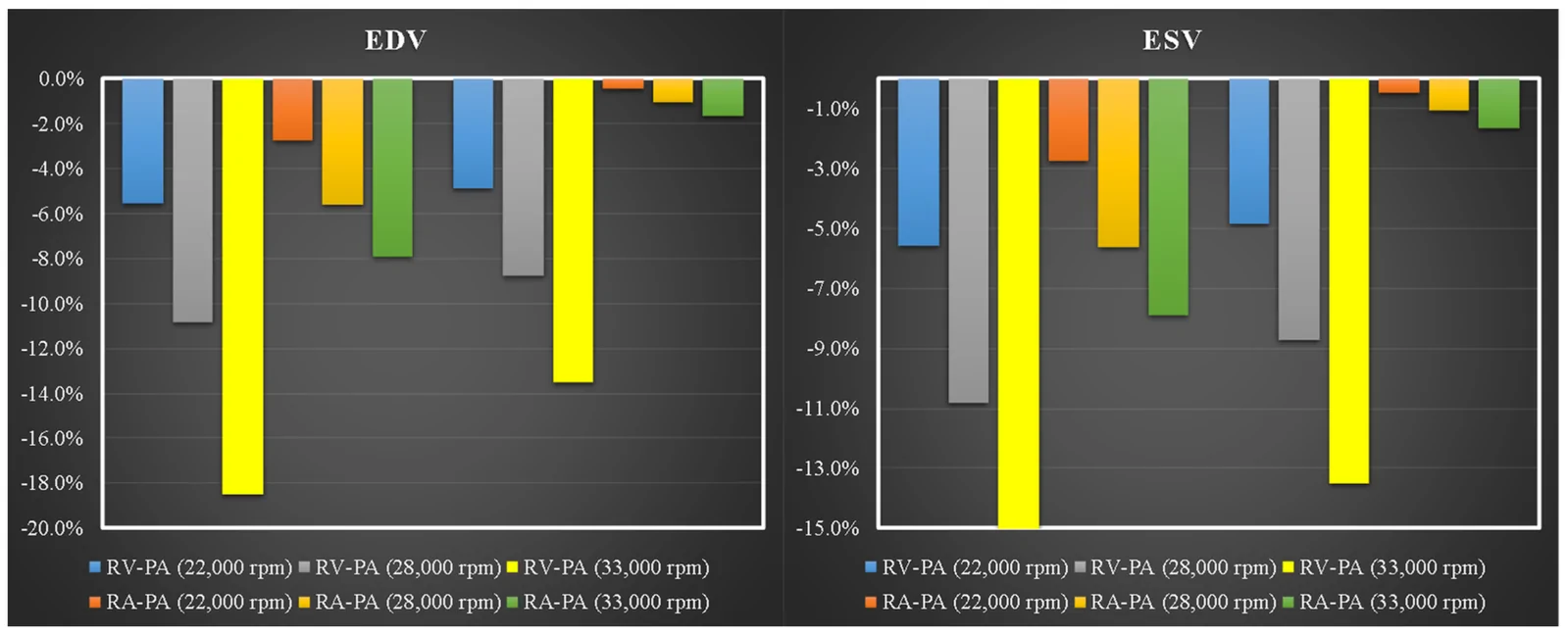
Percentage variations in right ventricular end diastolic volume (**left** panel) and right ventricular end systolic volume (**right** panel) in relation to the following starting conditions: systemic venous pressure (SVP) is kept constant at 15 [mmHg]; the wedge is set to 15 [mmHg]. The PAP was kept constant at 60 [mmHg]. Systemic venous resistance was kept constant at 30 [mmHg·cm^−3^·s].

**Figure 13:**
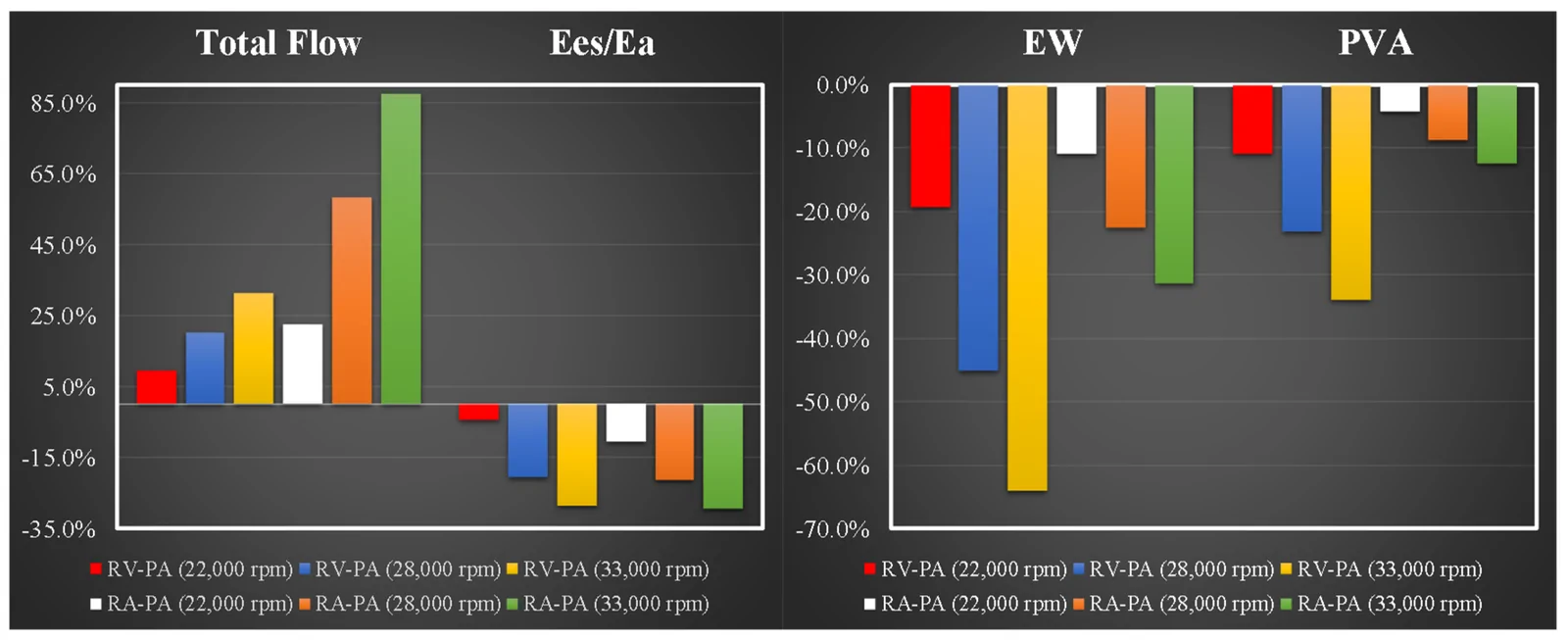
Percentage variations in TF and Ees/Ea (**left** panel) and EW and PVA (**right** panel) in relation to the following starting conditions: systemic venous pressure (SVP) is kept constant at 15 [mmHg]; PAWP is set to 15 [mmHg]. The PAP was kept constant at 60 [mmHg]. Systemic venous resistance was fixed at 30 [mmHg·cm^−3^·s].

**Figure 14:**
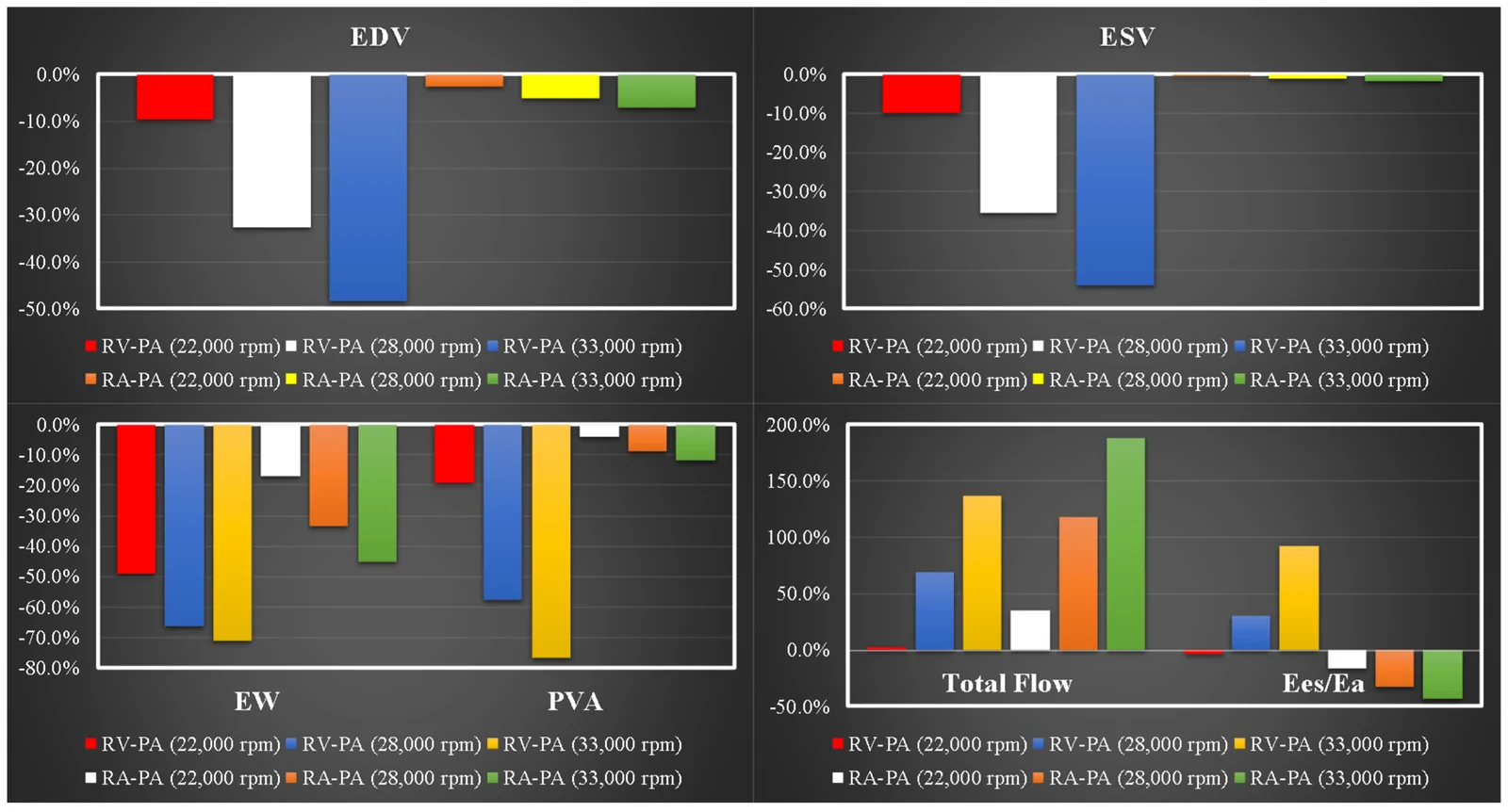
Percentage variations in right EDV (**left upper** panel), right ESV (**right upper** panel), EW and PVA (**left lower** panel) and total flow with Ees/Ea (**right lower** panel) in relation to the following starting conditions: systemic venous pressure (SVP) is kept constant at 10 [mmHg]; PAWP is set to [10 mmHg]. The PAP was kept constant at 60 [mmHg]. Systemic venous resistance was kept constant at 30 [mmHg·cm^−3^·s].

**Table 1:** Parameters used in Equation (7).

Rotary Pump Speed	*a*	*b*	*k*	R^2^
P1 flow [10,000 rpm]	−0.0051	−0.0091	1.1431	0.9832
P2 flow [13,000 rpm]	−0.0003	−0.0689	1.8413	0.9747
P3 flow [16,000 rpm]	0.0001	−0.0762	2.4316	0.9964
P4 flow [20,000 rpm]	−0.0002	−0.0511	3.016	0.9912
P5 flow [22,000 rpm]	−3 × 10^−6^	−0.0547	3.4257	0.9903
P6 flow [25,000 rpm]	6 × 10^−5^	−0.0541	4.0037	0.9914
P7 flow [28,000 rpm]	6 × 10^−5^	−0.0502	4.5514	0.9918
P8 flow [30,000 rpm]	6 × 10^−5^	−0.0492	4.9492	0.9925
P9 flow [33,000 rpm]	3 × 10^−5^	−0.0424	5.4355	0.9924

## Data Availability

The data presented in this study are available on request from the corresponding author due to specify restriction of Johnson & Johnson MedTech/Abiomed Europe GmbH.

## Appendix A

T_PB_	525	ms
T_PE_	750	ms
T_T_	60	ms
T_TE_	320	ms
E_rv,d_	5.265978 × 10^−2^	mmHg·cm^−3^
E_rv,s_	0.5	mmHg·cm^−3^
V_rv,0_	5.0	cm^3^
P_rv,0_	1.0	mmHg
E_ra,s_	0.123	mmHg·cm^−3^
E_ra,d_	2.4	mmHg·cm^−3^
V_ra,0_	2.0	cm^3^
P_ra,0_	1.0	mmHg
Rcp	0.0225	mmHg·cm^−3^·s
Rri	0.00755	mmHg·cm^−3^·s
Rinv	0.03733	mmHg·cm^−3^·s
Rro	0.00375	mmHg·cm^−3^·s
Rap	0.0878	mmHg·cm^−3^·s
Rvs	0.045	mmHg·cm^−3^·s
Lp	0.0000736	mmHg·cm^−3^·s^2^
Cp	4.8	mmHg^−1^·cm^3^
